# The Vertical Distribution of Sediment Archaeal Community in the “Black Bloom” Disturbing Zhushan Bay of Lake Taihu

**DOI:** 10.1155/2016/8232135

**Published:** 2016-01-17

**Authors:** Xianfang Fan, Peng Xing

**Affiliations:** ^1^State Key Laboratory of Lake Science and Environment, Nanjing Institute of Geography & Limnology, Chinese Academy of Sciences, Nanjing 210008, China; ^2^State Key Laboratory of Soil and Sustainable Agriculture, Institute of Soil Science, Chinese Academy of Sciences, Nanjing 210008, China

## Abstract

Using the Illumina sequencing technology, we investigated the vertical distribution of archaeal community in the sediment of Zhushan Bay of Lake Taihu, where the black bloom frequently occurred in summer. Overall, the Miscellaneous Crenarchaeotal Group (MCG), Deep Sea Hydrothermal Vent Group 6 (DHVEG-6), and* Methanobacterium* dominated the archaeal community. However, we observed significant difference in composition of archaeal community among different depths of the sediment. DHVEG-6 dominated in the surface layer (0–3 cm) sediment.* Methanobacterium* was the dominating archaeal taxa in the L2 (3–6 cm) and L3 (6–10) sediment. MCG was most abundant in the L4 (10–15 cm) and L5 (15–20 cm) sediment. Besides, DHVEG-6 was significantly affected by the concentration of total phosphorus (TP). And loss on ignition (LOI) was an important environmental factor for* Methanobacterium*. As the typical archaeal taxa in the surface layer sediment, DHVEG-6 and* Methanobacterium* might be more adapted to abundant substrate supply from cyanobacterial blooms and take active part in the biomass transformation. We propose that DHVEG-6 and* Methanobacterium *could be the key archaeal taxa correlated with the “black bloom” formation in Zhushan Bay.

## 1. Introduction

*Archaea* have traditionally been recognizable as extremophiles. However, culture-independent approaches such as 16S rRNA gene sequence analysis have shown* Archaea* can colonize vast reaches of the earth [1, 2]. Previous studies have extensively studied the methanogenic community in the freshwater lakes. And a review article indicates* Methanomicrobiales* and* Methanosarcinales* usually dominate the methanogenic community in freshwater sediment [3]. The uncultured archaeal groups of Miscellaneous Crenarchaeotic Group (MCG) and Deep Sea Hydrothermal Vent Group 6 (DHVEG-6) were also detected in freshwater lakes [4, 5]. The ubiquitous MCG is reported to contribute significantly to carbon and nitrogen cycling within the environments [6, 7]. DHVEG-6 was detected dominating in wastewater treating bioreactors [8], which indicated it might be heterotrophic and contribute to nutrient cycling. As a result of their diverse function and their ubiquity,* Archaea* may play a critical role in driving global biogeochemical cycles and maintaining the health of the freshwater environment.

“Black bloom” is a phenomenon in lakes, rivers, or seashores, which is characterized as hypoxic and malodorous [9, 10]. It often occurs during the summer after severe algae blooms and has become a serious ecological problem in water environments [11]. Lake Taihu is a large shallow eutrophic freshwater lake [12]. Serious cyanobacterial blooms frequently occurred in some lake zones of Lake Taihu, as a result of eutrophication [13]. As a result of cyanobacterial blooms, the “black bloom” happened frequently from 2007 to 2011 in Meiliang Bay, Gonghu Bay, and Zhushan Bay of Lake Taihu [14]. To date, researches on microbial diversity in the black bloom occurring lake zones have mainly focused on* Bacteria*.* Clostridium*,* Desulfovibrio,* and* Comamonadaceae* were found to be the main biological factor contributing to lacustrine black bloom [15, 16]. However, little is known about the diversity and vertical distribution of archaeal community in the lake sediment.

In this study, we investigated archaeal community composition in the sediment of the black bloom occurring area of Lake Taihu by using the next-generation sequencing method of Illumina. We particularly want to know whether the archaeal community composition is different among different layers of the sediment, given the difference in quality and quantity of organic materials among different sediment layers resulting from degradation of cyanobacterial blooms. We also want to see if there are some key sediment archaeal taxa contributing to the black bloom in Zhushan Bay.

## 2. Materials and Methods

### 2.1. Site Description and Sample Collection

Lake Taihu is a large shallow eutrophic lake with an area of 2338 km^2^ and an average depth of 1.9 m, located in the Yangtze River Delta (30°55.667′–31°32.967′N, 119°52.533′–120°36.167′E). Zhushan Bay is one of the most eutrophic bays in north of Lake Taihu, where the black bloom frequently occurred in summer.

Three sediment cores (8.6 cm inner diameter, 25 cm length) were collected from Zhushan Bay (31°23.705′N, 120°02.176′E) on July 9, 2010. The sediment cores were immediately transported to lab on ice. Once arriving at the laboratory, the sediment cores were sliced as 0–3 cm, 3–6 cm, 6–10 cm, 10–15 cm, and 15–20 cm. The three replicates for the five layers were mixed as thoroughly as possible. 0–3 cm, 3–6 cm, 6–10 cm, 10–15 cm, and 15–20 cm were labeled as L1, L2, L3, L4, and L5, respectively. Then, the five samples for DNA extraction were stored at −20°C and those for analysis of soil chemical properties at 4°C.

### 2.2. Physiochemical Analysis

The physicochemical properties of its overlying water were investigated using the YSI 550A instrument. Chlorophyll a (Chla) of sediment was determined using the HP8452 UV-Vis spectrophotometry. Total phosphorus (TP) was analyzed by molybdenum antimony resistance-colorimetric method. Total nitrogen (TN) was analyzed by Kjeldahl method. Total organic carbon (TOC) was analyzed by potassium dichromate oxidation-ferrous sulphate titrimetry method. Moisture content (MC) of sediment was measured after drying to constant weight at 105°C. Loss on ignition (LOI) was analyzed by heating at 550°C for 2 h. The TP, TN, TOC, MC, and LOI of sediment were analyzed according to Bao (2000) [17].

### 2.3. DNA Extraction, PCR Amplification, and Sequencing

Before DNA extraction, freeze-drying of the sediment samples at −53°C was performed in a freeze dryer (LABCONCO, 2.5 L). DNA was extracted from the five sediment layers in three replicates, using a FastDNA spin kit for soil (MP Biomedicals LLC, Ohio, USA). And according to the instruction, 0.5 g dried sediment was used for the DNA extraction. The quality and size of the DNA were checked by electrophoresis on 1% agarose gels.

The extracted DNA was amplified with the archaeal domain-specific primer set 519f (5′-CAGCMGCCGCGGTAA-3′) [18] with barcode, and 915r (5′-GTGCTCCCCCGCCAATTCCT-3′) [19]. Protocol and conditions for polymerase chain reaction (PCR) were according to Coolen et al. (2004) [20]. The amplicons were purified by Gel Extraction Kit (Takara Bio, Dalian, China). The size of amplicons was checked by electrophoresis on 2% agarose gels. The purity and quantity of amplicons were assessed using the Nanodrop ND-1000 UV-Vis Spectrophotometer (NanoDrop Technologies, Wilmington, DE, USA). Finally, the amplicons for different samples were pooled in equimolar ratios for sequencing analysis. Library preparation and DNA sequence analysis using an Illumina MiSeq paired-end 300 bp protocol (Illumina, Inc., San Diego, CA, USA) were performed at Shanghai BIOZERON Biotechnology Co., Ltd. (Shanghai, China). The raw sequence data was submitted to the ENA database (PRJEB10387).

### 2.4. Sequence Data Processing

Trimmomatic software was used to process the raw sequence data for quality control [21]. The PE reads were overlapped to assemble the final tag sequences with minimum overlap length as 10 bp. We removed all of the sequences that contained more than one ambiguous basis “N,” those that contained any errors in the forward or reverse primers, and those with more than 0.2 mismatch ratio within the overlap region. Tail base of reads with quality values below 20 was filtered, and the variable tags (overlapped length minus primers and barcodes) that were shorter than 50 bp were also removed. The obtained clean sequences were then analyzed at the Qiime platform [22]. The clean sequences were screened for chimeras using Usearch [23]. After the raw sequence data processing, sequences with length between 301 bp and 500 bp were used for the following analysis (see Figure S1 in Supplementary Material available online at http://dx.doi.org/10.1155/2016/8232135). Then, operational taxonomic unit (OTU) grouping was performed using Usearch software at 97% similarity. In order to filter the bacterial sequences during the assigning taxonomy process, an archaeal database was made by extracting archaeal sequences from the Silva database (release_119 http://www.arb-silva.de/). The database was provided by the Shanghai BIOZERON Biotechnology Co., Ltd. (Shanghai, China). Taxonomic data was then assigned to each representative sequence against the archaeal database at 97% similarity, using the RDP Classifier (http://sourceforge.net/projects/rdp-classifier/). Total of 17,358 sequences which cannot find affiliations were filtered out during this process.

### 2.5. Statistical Analysis

To perform the downstream analyses, the OTU matrix must be normalized to account for uneven sample sums. The normality of OTU data was performed using the command of “normalize.shared”, and the number of sequences for each sample was set as 19910, referring to the smallest group. The *α*-diversity of the archaeal community was indicated by the inverse Simpson diversity index. Besides, the sample coverage and the number of observed OTUs (Sobs) were also provided in Table 3. The normality of OTU data and analysis of the *α*-diversity of the archaeal community were performed using Mothur software [24]. The coverage percentage was calculated according to Good (1953) [25]. To further quantify observed difference, nonparametric statistics based on the Bray-Curtis dissimilarity index were performed using the OTU data. An analysis of similarities (ANOSIM) was performed to test if there was indeed a significant difference in archaeal community composition among different sediment layers. To visually interpret community dissimilarity and investigate the relationship between archaeal community data and physicochemical data, multivariate constrained ordination method was used. Detrended correspondence analysis (DCA) showed the largest axis length was 2.93 at OTU level and 1.69 at family level. Consequently, redundancy analysis (RDA) was selected, and the significance of total physicochemical factors was tested with Monte Carlo permutations (permu = 999). Environmental factors were selected by the functions of envfit (permu = 999) and vif.cca, and the environmental factors with *P* > 0.05 or vif > 20 were removed from the following analysis. The vif values of TOC, TN, and MC were higher than 20 and removed. The analyses of ANOSIM and RDA were conducted in *R* for statistical computing [26], using the vegan package [27]. The different distribution of the top 13 most abundant families among the five sediment layers was visually interpreted using cluster 3.0 [28]. The data, including the relative abundance of top 13 families, was log transformed and centered before analysis. And the color was set: contrast value was 2.0; positive value was set as red; negative value was set as green; zero value was set as white.

## 3. Results

### 3.1. Physicochemical Properties

The physicochemical properties of overlying water were showed in Table S1. Environmental parameters changed largely among the five sampling layers of Zhushan Bay sediment (Table 1). The average concentration values of all environmental parameters were observed much higher in the surface layers (L1-L2, 0–6 cm) than the deeper layers (L3–L5, 6–20 cm). The highest concentration values of Chla, TN, TOC, and LOI were observed in L2 (3–6 cm) sediment, while the concentration values of TP and MC were highest in L1 (0–3 cm) sediment.

### 3.2. Archaeal Community in Zhushan Bay Sediment

**Figure 1:**
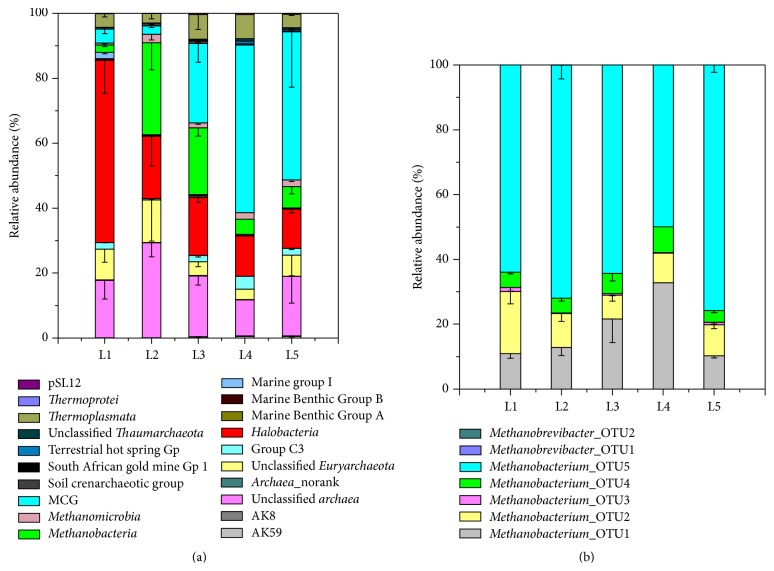
(a) Archaeal community composition of the five sediment layers at the class level (Miscellaneous Crenarchaeotic Group: MCG). Class* Methanobacteria* was included in this figure; (b) composition of* Methanobacteria* at OTU level; bars indicate the standard deviations of the technical replicates.

Results of taxonomic analysis indicated the top three archaeal classes were* Halobacteria* (24.42%), MCG (22.78%), and* Methanobacteria* (14.22%), except unclassified* Archaea* (Figure 1(a)). The archaeal community was dominated by* Halobacteria* in the L1 (0–3 cm) sediment. In the L2 (3–6 cm) sediment, methanogen (*Methanobacteria* and* Methanomicrobia*) was main component of archaeal community. In the L3 (6–10 cm) sediment, the relative abundance of MCG obviously increased. And in the L4 (10–15 cm) and L5 (15–20 cm) sediment, MCG dominated in the archaeal community.

As the dominating methanogen,* Methanobacteria* consisted of seven OTUs (Figure 1(b)). These seven OTUs were affiliated to the genera of* Methanobacterium* and* Methanobrevibacter*. The composition of* Methanobacteria* did not change significantly among the five sediment layers. And* Methanobacterium*_OTU5 was the most abundant OTU in all of the five sediment layers.

Additionally, MCG_norank, DHVEG-6, and* Methanobacteriaceae* were the top three dominating families in the sediment, except unclassified* Archaea* (Table 2). DHVEG-6 was affiliated to* Halobacteria*, while* Methanobacteriaceae*  was affiliated to* Methanobacteria*.

### 3.3. Statistical Analysis

Coverage estimate indicated that the archaeal 16S rRNA gene libraries for each sample were large enough to capture the total estimated OTUs (Table 3). And the average values of inverse Simpson index suggested that the L2 (3–6 cm) sediment harbored the lowest diversity of archaeal community, while the highest diversity of archaeal community was found in the L1 (0–3 cm) sediment.

**Figure 2:**
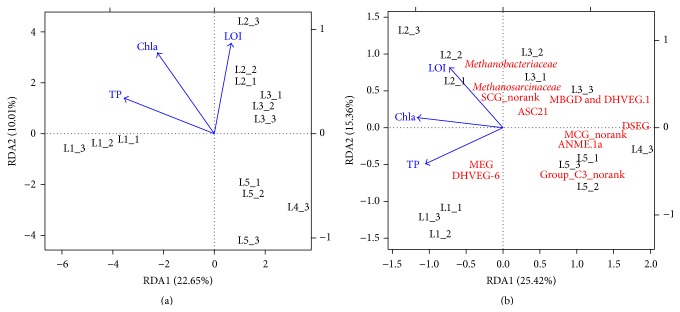
(a) The relationship of archaeal community composition among the five sediment layers with the major environmental factors at the OTU level. Redundancy analysis (RDA) plot was drawn by RDA1 and RDA2. (b) The most abundant 13 families were correlated with the major environmental factors (MCG: Miscellaneous Crenarchaeotic Group; DHVEG-6: Deep Sea Hydrothermal Vent Gp-6; MEG: Miscellaneous Euryarchaeotic Group; DSEG: Deep Sea Euryarchaeotic Group; MBGD: Marine Benthic Group D; ANME: anaerobic methanotroph).

Global ANOSIM comparison indicated the overall archaeal community composition among the five sediment layers was significantly different (*R* statistic = 0.932, *P* = 0.001). Observed difference was qualitatively displayed in the RDA plot, where relative similarities among sediment layers were presented by clusters of layer-specific data points (Figure 2(a)). Besides, result of RDA at the OTU level showed the first axis explained 22.65% of total microbial variance and the second axis 10.01%. The Monte Carlo permutation test at the OTU level showed the environmental factors were significantly related to archaeal community distribution (Pseudo-*F* = 1.93; significance level = 0.001). Envfit test at the OTU level suggested Chla and TP were significant at the 0.001 level (*P* = 0.001), while LOI was significant at the 0.05 level (*P* = 0.002). The RDA plot at the OTU level indicated TP was a more important factor for the archaeal community in the L1 sediment, while LOI was a more important factor for the archaeal community in the L2 and L3 sediment. Archaeal community in the L1 and L2 sediment was both affected by the Chla. Moreover, result of RDA at the family level showed the first axis explained 25.42% of total microbial variance and the second axis 15.36% (Figure 2(b)). The Monte Carlo permutation test at the family level showed the environmental factors were significantly related to archaeal community distribution (Pseudo-*F* = 2.98; significance level = 0.001). Envfit test at the family level suggested Chla and TP were significant at the 0.001 level (*P* = 0.001), while LOI was significant at the 0.05 level (*P* = 0.004). The RDA plot at the family level indicated TP was an important factor for DHVEG-6 and MEG, while LOI was an important factor for* Methanobacteriaceae*,* Methanosarcinaceae*, and SCG_norank.

**Figure 3:**
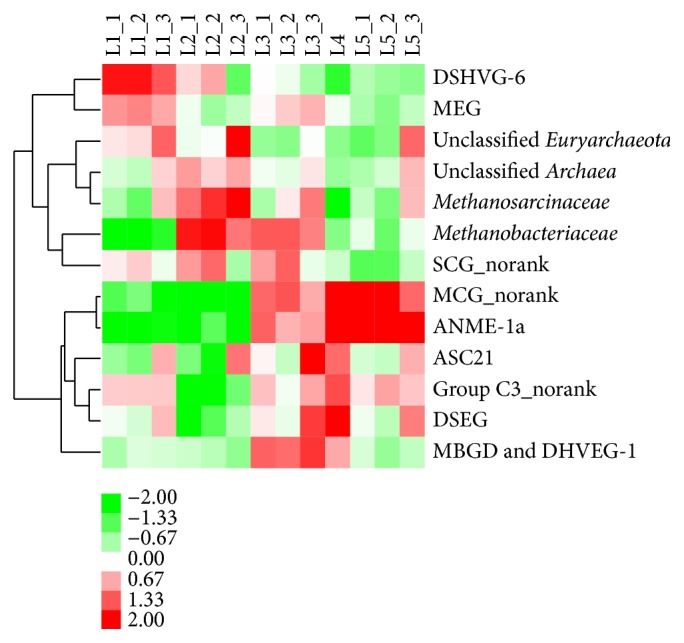
Clustering analysis of the most abundant 13 families versus different sediment layers (MCG: Miscellaneous Crenarchaeotic Group; DHVEG-6: Deep Sea Hydrothermal Vent Gp-6; MEG: Miscellaneous Euryarchaeotic Group; DSEG: Deep Sea Euryarchaeotic Group; MBGD: Marine Benthic Group D; ANME: anaerobic methanotroph).

Cluster analysis of the top 13 most abundant families identified specific families; those were differentially distributed among the five sediment layers (Figure 3). DHVEG-6 was most abundant in the L1 (0–3 cm) sediment.* Methanobacteriaceae* and* Methanosarcinaceae* were most abundant in the L2 (3–6 cm) sediment.* Methanobacteriaceae* was more abundant in the L3 (6–10 cm) sediment, compared with the ones in L1, L4, and L5 sediment. Group C3_norank and Deep Sea Euryarchaeotic Group (DSEG) were most abundant in the L4 (10–15 cm) sediment. MCG_norank and anaerobic methanotroph (ANME)-1a were most abundant in the L4 (10–15 cm) and L5 (15–20 cm) sediment.

## 4. Discussion

### 4.1. Significant Vertical Heterogeneity of Archaeal Community in Zhushan Bay Sediment

Significant difference of overall archaeal community composition among the five sediment layers was found (*P* = 0.001), which may result from the decreasing nutrients from the surface layers to the deep layers (Figure 2). The vertical heterogeneity of archaeal community was also observed in another lake zone of Lake Taihu [29].

DHVEG-6 differentially distributed among the five sediment layers and was most abundant in L1 sediment (Figure 3). DHVEG-6 was known as haloarchaea previously, as it had been detected in hydrothermal sediment [30], deep sea methane seep sediment [31], hypersaline [32], and shallow saline [33] lakes. However, DHVEG-6 was also detected in the water of freshwater lake [5] and in municipal wastewater treating methanogenic bioreactors [34]. Moreover, it is found that DHVEG-6 was the predominant uncultured archaeal community in wastewater treatment sludge, being most abundant in the nitrogen-/phosphate-removing wastewater treatment sludge [8]. This might indicate some groups of DHVEG-6 were more adaptive to high substrate supply, and phosphate might be a key factor to it. And the result of this study also indicated members of DHVEG-6 were significantly affected by TP (Figure 2(b)). More organic matters and higher level of TP in the surface sediment may favor the DHVEG-6.

*Methanobacteriaceae* differentially distributed among the five sediment layers and was most abundant in L2 and L3 sediment (Figure 3). Besides,* Methanosarcinaceae* was also the most abundant family in the L2 sediment (Figure 3). However, previous studies revealed that the* Methanomicrobia* usually dominated the methanogenic communities in freshwater sediment, while the* Methanobacteria* occurred scarcely [3], using clone libraries and/or fluorescent in situ hybridization methods.* Methanobacteriaceae *was usually found to be the predominant methanogen in the wastewater treatment sludge or municipal solid waste landfill [35]. It could be noted that strains and type strains of* Methanobacterium* and* Methanosarcinaceae* were commonly cultivated from freshwater lakes by using media with high substrate concentrations [36, 37]. And in this study, 99.99% sequences of* Methanobacteriaceae* were affiliated to* Methanobacterium* (Figure 1(b)). These indicated* Methanobacterium* and* Methanosarcinaceae* were more adaptive to high carbon concentrations. And the result of this study also indicated members of* Methanobacterium* and* Methanosarcinaceae* were significantly affected by LOI (Figure 2(b)). Serious cyanobacterial blooms in Zhushan Bay provide abundant labile organic matters and may benefit the* Methanobacterium* and* Methanosarcinaceae.*

Group C3_norank and DSEG were most abundant in the L4 sediment (Figure 3). Group C3 and DSEG have been mostly detected in marine sediment [38–40]. Moreover, in oxygen-depleted zones of a deep lake, a high number of 16S rRNA transcripts were associated with Group C3, which indicated the potential for this uncharacterized group to contribute to nutrient cycling in lakes [5]. In Lake Redon, DSEG bloomed in deep stratified waters both in summer and in early spring, and a positive and significant relationship was found between DSEG and putative ammonia oxidizing* Thaumarchaeota* [41]. ANME-1a and MCG were most abundant in the L4 and L5 sediment (Figure 3). ANME-1 was always found to be loosely associated with sulfate-reducing bacteria [42]. And in the freshwater sediment, it was often detected in the sulfate methane transition zones [43, 44]. During the black bloom occurring, high concentration of H_2_S and a great amount of sulphate-reducing* Bacteria* were found in the sediment and water of Zhushan Bay [16, 45]. And the ANME-1a might benefit from the black bloom in Zhushan Bay. Members of the highly diverse MCG are globally distributed in various marine and continental habitats [46]. Up to now, no isolate of MCG has been cultivated or characterized. However, previous studies suggested that MCG was anaerobic heterotrophs and did not participate in methane and sulfur cycles but likely used refractory organic carbon present in deeper sediment of Zhushan Bay, such as detrital proteins and aromatic compounds [7, 47, 48].

### 4.2. DHVEG-6 and Methanobacterium Might Be the Key Sediment Archaeal Taxa Contributing to the Black Bloom in Zhushan Bay

Cyanobacterial blooms have become common in inland bodies of water, including freshwater lakes, due to the overloading of phosphorus and nitrogen [49]. The accumulation and breakdown of large amount of cyanobacterial biomass easily result in the depletion of dissolved oxygen, which often leads to hypoxia and “black bloom” in lakes [11]. Previous studies have indicated the presence of novel bacterial groups and have suggested that the bacterial groups varied temporally with the concentrations of oxygen and phosphate within hypoxic zones in lakes and marine [16, 50, 51]. However, no reports are found studying archaeal community in the “black bloom” occurring zones. In this study, DHVEG-6 and* Methanobacterium *were dominating archaeal groups in the surface sediment layers (0–10 cm), which was most affected by the cyanobacterial blooms or “black bloom” (Table 2, Figure 3). DHVEG-6 and* Methanobacterium* were found adapted to the habitats with high substrate supply, such as wastewater treatment sludge [8, 35]. Besides, DHVEG-6 was significantly affected by the concentration of phosphate, and labile organic matters were an important environmental factor for* Methanobacterium* (Figure 2(b)). Moreover, members of* Methanobacterium* were strictly anaerobic* Archaea *[36]. However, more work is required to enable a better understanding of the roles of DHVEG-6 in Zhushan Bay sediment. And the dominating uncultured species of* Methanobacterium_*OTU5 (Figure 1(b)) was expected to be further understood by isolation and culture methods. DHVEG-6 and* Methanobacterium *might actively take part in the degradation of cyanobacterial biomass, contributing to the black bloom in Zhushan Bay.

## 5. Conclusion

This is the first study to investigate the vertical distribution of archaeal community in a “black bloom” disturbing area using high throughput sequencing technology. Our work revealed that the vertical distribution of archaeal community in the 0–20 cm sediment was significantly heterogeneous. DHVEG-6 and* Methanobacterium *dominated in the surface sediment, which might be the key archaeal taxa correlated with the “black bloom” occurrence in Zhushan Bay, Lake Taihu. This work shed light on the contribution of* Archaea* to the black bloom formation in this high-risk area of cyanobacterial blooms. More work is needed to get a better understanding of the roles of DHVEG-6 and uncultured* Methanobacterium* in the fast nutrient cycling in the surface sediment of this area.

## Supplementary Material

Figure S1 showed the length distribution of the clean sequences; Figure S2 showed the differences of archaeal community composition among the five sediment layers at OTU level in NMDS plot; Table S1 showed the main characteristics of the overlying water at the sampling site (water depth, DO, pH, ORP, Turbidity).

## Figures and Tables

**Table 1:** Some physicochemical characteristics of the five sediment layers.

Sample	Chla (*μ*g/kg)	TP (g/kg)	TN (g/kg)	TOC (g/kg)	LOI (%)	MC (%)
L1	616.99	1.13	1.23	12.35	2.15	51.08
L2	704.84	0.93	1.35	13.54	6.21	48.74
L3	309.51	0.59	0.83	8.32	2.47	47.49
L4	110.86	0.53	0.86	8.64	2.18	46.92
L5	159.64	0.66	0.82	8.25	1.26	44.08

**Table 2:** The top 13 most abundant families in all of the samples from the five sediment layers.

Class	Family	Percentage (%)
MCG	MCG_norank	22.78
*Halobacteria*	DHVEG-6	20.10
Unclassified *Archaea*	Unclassified *Archaea*	19.94
*Methanobacteria*	*Methanobacteriaceae*	14.22
Unclassified *Euryarchaeota*	Unclassified *Euryarchaeota*	7.50
*Halobacteria*	MEG	2.36
Group C3	Group C3_norank	1.80
*Thermoplasmata*	ASC21	1.54
*Halobacteria*	DSEG	1.33
*Thermoplasmata*	MBGD and DHVEG-1	1.12
*Methanomicrobia*	ANME-1a	0.58
*Methanomicrobia*	*Methanosarcinaceae*	0.54
SCG	SCG_norank	0.53

*Note*. “Percentage” was the average value of all samples; Miscellaneous Crenarchaeotic Group (MCG); Deep Sea Hydrothermal Vent Gp-6 (DHVEG-6); Miscellaneous Euryarchaeotic Group (MEG); Deep Sea Euryarchaeotic Group (DSEG); Marine Benthic Group D (MBGD); anaerobic methanotroph (ANME).

**Table 3:** *α*-Diversity analysis for archaeal community of the five sediment layers (standard deviations of replicates are in brackets).

Sample	Coverage (%)	Sobs	Inverse Simpson
L1	98.00 (0.15)	1641 (134)	83.19 (7.96)
L2	99.25 (0.09)	809 (177)	15.70 (6.49)
L3	99.17 (0.47)	891 (310)	30.11 (3.90)
L4	99.60	798	63.35
L5	98.62 (0.42)	1212 (363)	54.78 (15.92)
