# Supplementary material for: The Vertical Distribution of Sediment Archaeal Community in the “Black Bloom” Disturbing Zhushan Bay of Lake Taihu
Source: Archaea. 2016 Jan 17;2016:8232135. doi: 10.1155/2016/8232135 (PMC4738990; doi:10.1155/2016/8232135)
Supplement: Supplementary file 1 — Figure S1 showed the length distribution of the clean sequences; Figure S2 showed the differences of archaeal community composition among the five sediment layers at OTU level in NMDS plot; Table S1 showed the main characteristics of the overlying water at the sampling site (water depth, DO, pH, ORP, Turbidity). [file 8232135.f1.docx]

Supplement materials

Figure S1 Length distribution of the clean sequences

Figure S2: Differences of archaeal community composition among the five sediment layers was displayed in NMDS.

Table S1: Main characteristics of the overlying water at the sampling site.

| Index | Water depth(m) | DO  (mg/L) | pH | ORP(mV) | Turbidity (NTU) |
| --- | --- | --- | --- | --- | --- |
| Mean value | 1.186 | 8.192 | 8.668 | 123 | 72.22 |
